# Usability of an Immersive Augmented Reality Based Telerehabilitation System with Haptics (ARTESH) for Synchronous Remote Musculoskeletal Examination

**DOI:** 10.5195/ijt.2019.6275

**Published:** 2019-06-12

**Authors:** ALEKS BORRESEN, CODY WOLFE, CHUNG-KUANG LIN, YUAN TIAN, SURAJ RAGHURAMAN, KLARA NAHRSTEDT, BALAKRISHNAN PRABHAKARAN, THIRU ANNASWAMY

**Affiliations:** 1 THE UNIVERSITY OF TEXAS SOUTHWESTERN MEDICAL CENTER, DALLAS, TX, USA; 2 THE UNIVERSITY OF TEXAS AT DALLAS, RICHARDSON, TX, USA; 3 UNIVERSITY OF ILLINOIS, URBANA-CHAMPAIGN, IL, USA; 4 VA NORTH TEXAS HEALTH CARE SYSTEM, DALLAS, TX, USA

**Keywords:** Augmented-reality, Haptics, Telerehabilitation, Telemedicine

## Abstract

This study describes the features and utility of a novel augmented reality based telemedicine system with haptics that allows the sense of touch and direct physical examination during a synchronous immersive telemedicine consultation and physical examination. The system employs novel engineering features: (a) a new force enhancement algorithm to improve force rendering and overcoming the “just-noticeable-difference” limitation; (b) an improved force compensation method to reduce the delay in force rendering; (c) use of the “haptic interface point” to reduce disparity between the visual and haptic data; and (d) implementation of efficient algorithms to process, compress, decompress, transmit and render 3-D tele-immersion data. A qualitative pilot study (n=20) evaluated the usability of the system. Users rated the system on a 26-question survey using a seven-point Likert scale, with percent agreement calculated from the total users who agreed with a given statement. Survey questions fell into three main categories: (1) ease and simplicity of use, (2) quality of experience, and (3) comparison to in-person evaluation. Average percent agreements between the telemedicine and in-person evaluation were highest for ease and simplicity of use (86%) and quality of experience (85%), followed by comparison to in-person evaluation (58%). Eighty-nine percent (89%) of respondents expressed satisfaction with the overall quality of experience. Results suggest that the system was effective at conveying audio-visual and touch data in real-time across 20.3 miles, and warrants further development.

Since its inception, telerehabilitation has grown to encompass disciplines across the medical spectrum. This includes fields ranging from clinical and post-surgical cardiopulmonary care (Lundell, Holmner, Rehn, Nyberg, & Wadell, 2015; Segrelles Calvo et al., 2014), to COPD (Lundell et al., 2015; Segrelles Calvo et al., 2014), orthopedic repairs (Agostini et al., 2015; Cottrell, Galea, O’Leary, Hill, & Russell, 2017), speech-language pathology, and importantly, neurological impairment (Agostini et al., 2015; Amatya, Galea, Kesselring, & Khan, 2015; Chen et al., 2015; Iruthayarajah, McIntyre, Cotoi, Macaluso, & Teasell, 2017). These disciplines have each demonstrated opportunities to make use of telerehabilitation technology. Progressively, more of these needs are being met with novel technology; development in virtual environment creation and haptic technology have been particularly important to recent advancement in telerehabilitation (Piggott, Wagner, & Ziat, 2016).

Virtual reality can be described as a medium through which humans can visualize, manipulate, and interact with computers and extremely complex data (Aukstakalnis, Blatner, & Roth, 1992), and allows the user to “become immersed within computer-generated environments” (Rizzo, 1997, p. 1). Its efficacy and application to telerehabilitation have been extensively reviewed (Burdea, 2003; Holden, 2005; Howard, 2017; Larson, Feigon, Gagliardo, & Dvorkin, 2014). Virtual reality’s success in improving clinical outcomes has been enhanced by the integration of haptic feedback technology (Srinivasan & Basdogan, 1997).

Srinivasan and Basdogan (1997) described haptic technology by referring to its ability to allow physical interaction with environments, real or virtual. This interaction includes both the user’s manipulation of the environment and the environment providing feedback to the user via a haptic device. Maximizing the feeling of being a part of the virtual environment is known as “telepresence,” a key goal in designing this technology. Doing so enhances the quality of the experience, allowing for effective remote interactions. Telepresence has been used in remote delivery of rehabilitation in healthcare, but primarily through robots (Koceski & Koceska, 2016; O’Carroll, Hentz, Aguilar, & Demaerschalk, 2015). There are no published reports of synchronous telerehabilitation delivered via telepresence.

Another related technology is augmented reality. As opposed to immersing oneself in a virtual environment, augmented reality systems overlay digital images onto real-world environments. In doing so, the system augments real-world information with digital information (e.g., pictures, videos, instructions, clinical data) enhancing the experience. Similar to the combination of virtual reality systems with haptic machines, augmented reality systems can also work with haptic devices to monitor, resist, direct, or otherwise influence or be influenced by the user’s movements. The systems’ capacity for realistic interaction makes them viable targets for research into therapeutic purposes. In addition, augmented reality systems typically allow a user’s facial features to remain unobstructed, making natural face-to-face interaction easier than with other virtual reality systems. Reports on the utility of immersive virtual environments and haptic feedback in post-stroke rehabilitation have been previously published (Piggott et al., 2016; Shull & Damian, 2015; van Delden, Peper, Kwakkel, & Beek, 2012).

These novel technologies have had a demonstrable positive effect on rehabilitation (Burdea, 2003; Holden, 2005; Howard, 2017; Larson et al., 2014). One major benefit to their use is the increase in data that providers can receive about patients through the machine. Augmented reality systems can allow the provider to visually monitor patients’ performance, and haptic machines can record quantitative information while they exercise. Some studies have even demonstrated equal and superior performances in the rehabilitation regimens employing these technologies as compared to traditional rehabilitation routines (Howard, 2017). It has been shown, thus, that the technologies used in telerehabilitation exercise regimens can, at least in some ways, be compared to in-person rehabilitative therapy (Cottrell et al., 2017). One notable exception is the information garnered from physical examination. In-person examination of patients is central to assessments tracking their progress. The element of touch is lost during typical telemedicine consultations. To date, employing augmented reality and haptic technologies to conduct a physical exam has not been demonstrated.

Current telemedicine physical examinations are limited to what is visible via video. Moreover, physical exam maneuvers require a healthcare provider at the patient’s location who physically interacts with the patient and reports findings to the primary site. A proposed solution is that sophisticated haptics may allow for the remote conduct of a full physical examination, including palpation or evaluation of tenderness, range of motion, strength, tone, reflexes, and sensation. In a cautionary vein, the deployment of this novel, problem-solving technology could present new challenges. First, there are opportunities for miscommunication. This is especially true if the healthcare providers are interacting for the first time, complicating communication and trust. Second, a healthcare provider in a remote location may not be used to seeing patients with the condition for which the telehealth consult was placed (assuming tele-consultation is taking place due to a lack of expertise in a given patient’s condition).

The purpose of this study was to assess the usability of a novel **A**ugmented **R**eality based **TE**lerehabilitation **S**ystem with **H**aptics (**ARTESH**), to physically examine patients with upper extremity complaints remotely. After using the telerehabilitation system, users at the patient and physician sites were surveyed regarding utility, ease of use, reliability, and satisfaction.

## MATERIALS & METHODS

**Figure 1:**
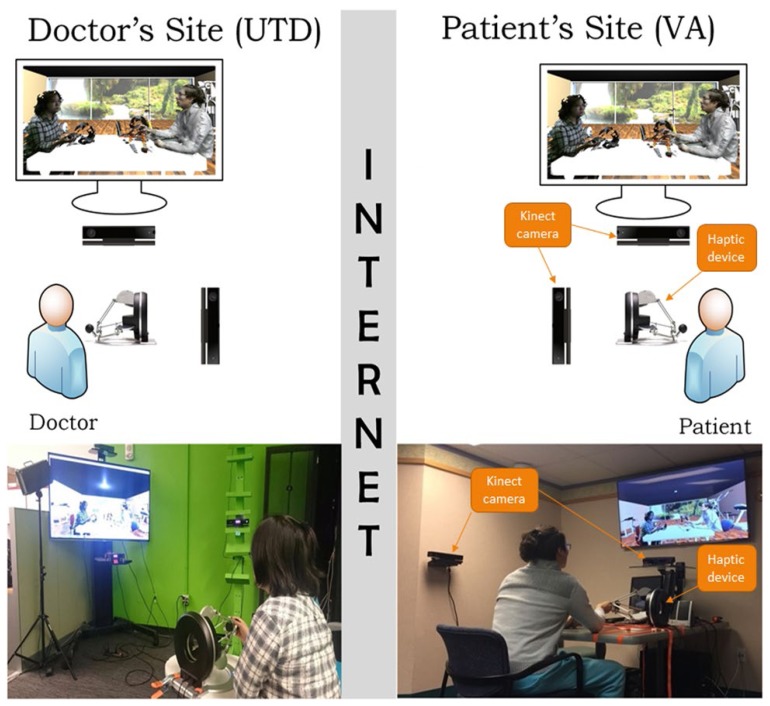
Each site was equipped with two Xbox Kinect RGB-D cameras (arranged orthogonal to user), one haptic controller (fastened to the desk), a 3D-capable TV, active 3D-glasses, and a computer. The computers were networked via the internet, and audio, video, and force data were transmitted in real-time between the two sites.

**Figure 2:**
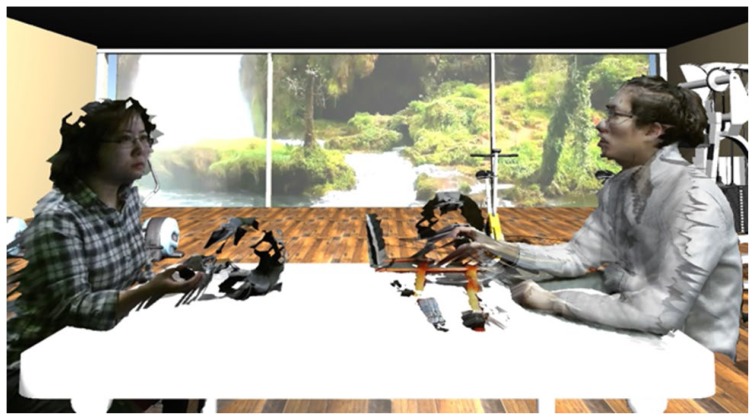
2D-Example of the 3D-video which the patient and evaluator see during the patient interaction. 3D-Glasses were necessary in order to perceive the three-dimensional video of the users/environment. (The artifacts seen around the edges were stitching artifacts generated when the two depth-sensing camera images were stitched together.)

The **A**ugmented **R**eality based **TE**lerehabilitation **S**ystem with **H**aptics (**ARTESH**) is a telerehabilitation system which uses haptic feedback and depth sensing camera technology (Red-Green-Blue-Depth/RGB-D cameras) to allow a clinician and a patient to interact remotely through video, audio, and touch. This is achieved using two orthogonal depth-sensing cameras that record 3D video, and remotely paired haptic controllers that transmit forces between the two locations. See Figure 1 for a visual depiction and description of the setup at each site. The two sites were networked together via the internet, and camera and haptic data from both sites were used to render a computer-generated therapy environment as seen in Figure 2. The environment was rendered in the Unity® game engine and allowed for any desired therapy environment and camera angles to be implemented. Technical details on the computer science and engineering behind **ARTESH** have been published elsewhere (Tian et al., 2017; Venkatraman et al., 2014). In brief, the ARTESH system enabled bi-directional force feedback and motion, facilitating synchronous remote physical evaluation with audio, visual and haptic data transmitted live over the internet. The system employed the following novel engineering features in design: (a) a new force enhancement algorithm to improve force rendering and overcoming the “just-noticeable-difference” limitation; (b) an improved force compensation method to reduce the delay in force rendering; (c) use of the “haptic interface point” to reduce disparity between the visual and haptic data; and (d) implementation of efficient algorithms to process, compress, decompress, transmit and render 3-dimensional tele-immersion data. The images thus rendered were displayed on a 3D-TV and users wore 3D-glasses in order to view the interaction occurring as seen in Figure 2. The haptic device provided force feedback to allow the user to feel the actions of the other remote user.

The maximum force transmitted by the system was capped below the maximum potential output force of 12 Newtons, deliverable by the haptic device. This was done to prevent potential injury in the event of a positive force feedback loop developing between the two haptic devices. 1

**ARTESH** was used to assess upper extremity function in a set of twenty participants. The sample size (n=20) was estimated from similar telerehabilitation evaluation projects reported in the literature (Schutte et al., 2012). Initially, five healthy physician volunteers without any history of upper limb problems were recruited from the Physical Therapy and Physical Medicine and Rehabilitation (PM&R) telerehabilitation personnel. They evaluated the system and provided usability information from a clinical perspective. Fifteen subjects referred to hospital-based PM&R clinic with a chief complaint of arm and/or shoulder pain and/or weakness, were recruited for the study. Inclusion criteria were: patients who were referred to the PM&R physician, physical therapy clinic, or occupational therapy clinic for initial evaluation and management of upper limb disorders. Exclusion criteria included inability to participate in a physical examination of the upper limb, due to severe weakness or pain.

After a patient agreed to participate in the study and provided written informed consent and a focused history, they were scheduled a date for both “in-person” and “remote” evaluations. During this evaluation, the patients were taken through a series of range of motion (ROM) and maximum isometric strength (MIS) assessments of their affected extremity. After ROM and MIS results as well as reports of pain were recorded, patients filled out a modified version of the Telehealth Usability Questionnaire (Parmanto, Lewis, Graham, & Bertolet, 2016; Parmanto et al., 2010). All participants initially had the “in-person” evaluation, following which they underwent the “remote” evaluation. Each clinician was blinded to the other clinician’s findings until after the study. Each clinical evaluation and survey completion took about 40 minutes total, or 20 minutes each.

There was no randomization of the patients as all participants went through the same evaluations. The two clinicians were two of the authors of this study (AB and TA). The in-person evaluator was trained, licensed, and credentialed to operate the **ARTESH** system, and trained the remote evaluator to operate the **ARTESH** system. The remote evaluator evaluated the patient only remotely, thereby minimizing potential bias.

After collecting survey data from the five healthy physician volunteers, fifteen additional questions were added to the Telehealth Usability Questionnaire, for additional feedback regarding system performance. (For these fifteen additional questions, data is only available for fifteen of the twenty study subjects.) The modified Telehealth Usability Questionnaire consisted of 26 questions regarding usability, usefulness, ease of use, reliability, and satisfaction. Questions were rated on a 7-point Likert scale ranging from: (1) “completely disagree,” to (4) “neither agree nor disagree,” to (7) “completely agree,” or (NA) “not applicable.” The questionnaire was used to gather data to enable comparisons between user satisfaction for the telerehabilitation system versus the standard in-person clinical assessment. Survey results were used to facilitate adjustments to enhance the system before assessing its implementation in regular clinical practice. The questionnaire also contained a comments section where participants could express opinions not covered explicitly by the questionnaire.

This was a small pilot study and statistical significance was therefore not calculated. Instead, qualitative statistics were used to show trends in the survey data. For example, the number of respondents who answered “weakly agree” (5), “agree” (6), or “strongly agree” (7) were pooled and divided by the total number of respondents to calculate a “percent agree” statistic, which denoted the percentage of study participants that agreed with a given statement in the questionnaire.

This research was approved by our institution’s committee on research ethics (Institutional Review Board) and was conducted in accordance with the Declaration of the World Medical Association.

## RESULTS

Seven questions were asked within the category of “ease and simplicity.” When examined in aggregate these seven questions averaged 5.97 of 7 (SD=1.36, Range=1–7) on the Likert scale, implying an overall agreement with statements categorizing the system as easy to use. This corresponded to a positive response from 85.9% of users (see **f**igure 3). Specifically, users agreed that the system was easy to use (93%) and learn (85%), that there was sufficient clarity of instruction (85%), and that they were able to efficiently complete their evaluation using this system (85%). Additionally, most users felt comfortable using the system (85%), and 80% agreed that it was “nice to look at.” Almost all users (90%) reported that they were overall satisfied with how easy it was to use the system.

**Figure 4:**
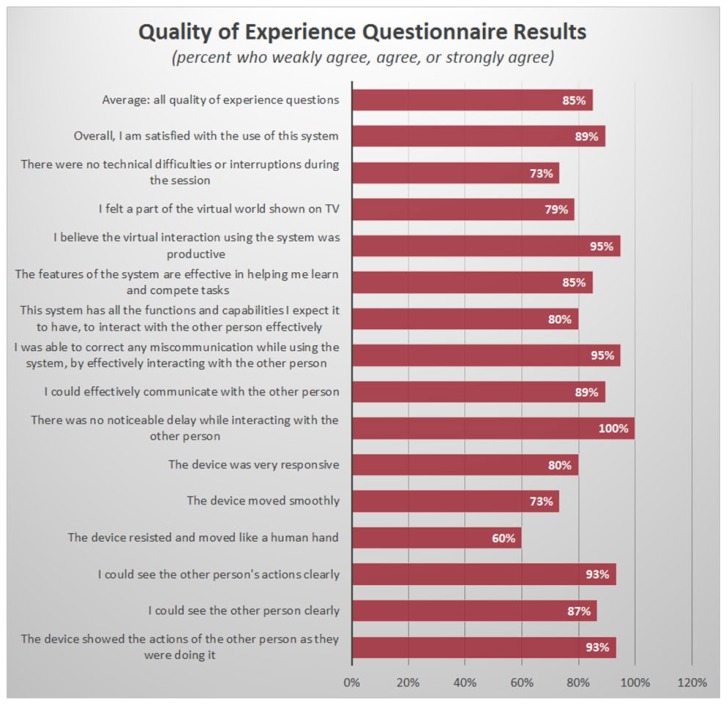
Results of “Quality of Experience” section of telehealth usability questionnaire reported as percent of respondents who rated > 4 on a 7-point Likert scale (corresponding to “weakly agree”, “agree”, or “strongly agree”).

The “quality of experience” section of the questionnaire was comprised of fifteen questions that attempted to assess user experience. When responses for this section were taken in aggregate, the system rated 5.88 of 7 (SD=1.34, Range=1–7). This corresponded to a positive response from 84.9% of users (see Figure 4). These results suggested that users perceived that they were able to: see the other person clearly (87%), effectively communicate with the other person (89%), and correct any miscommunication by effectively interacting with the other person through the telerehabilitation system (95%). With regard to haptics, users felt that the device was responsive (80%), moved smoothly (73%), and resisted and moved “like a human hand” (60%). All users reported no noticeable delay while interacting with the other person (100%) and most reported no technical difficulties or interruptions during their sessions (73%). When asked if users “felt part of the virtual world” the majority agreed (79%), and almost all users thought the interaction was productive (95%). When asked if users were satisfied with the overall quality of experience, 89% of respondents agreed. Together this suggested that the system was effective at conveying audio-visual and touch data in real-time across 20.3 miles.

**Figure 5:**
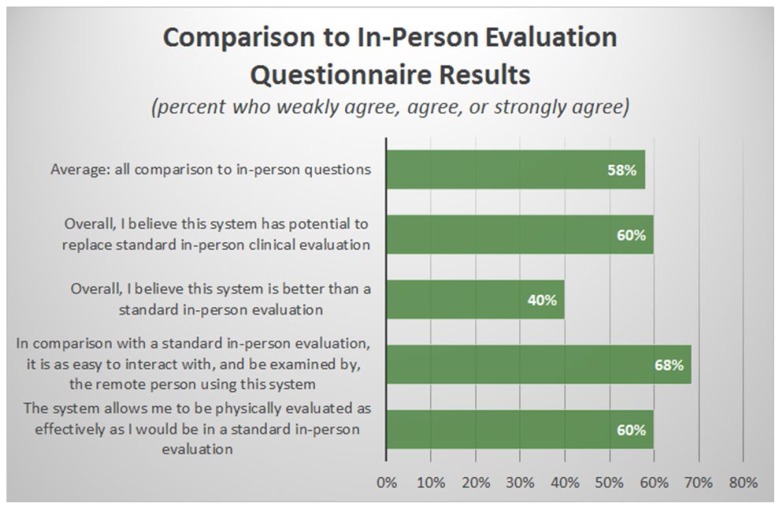
Results of “Comparison to In-person Evaluation” section of telehealth usability questionnaire reported as percent of respondents who rated > 4 on a 7-point Likert scale (corresponding to “weakly agree”, “agree”, or “strongly agree”).

The “comparison to in-person evaluation” section of the questionnaire was comprised of 4 questions. This was the lowest scoring section of the questionnaire with an average overall rating of 4.81 of 7 (SD=2.16, Range=1–7), which corresponded to a positive response from only 58% of the users (see Figure 5). For example, in response to the question of whether this system is better than a standard in-person evaluation, only 40% of users agreed. This corresponded to an average Likert of 3.93 (SD=1.98, Range=1–7), indicating that users slightly disagreed with the previous statement. Interestingly, when asked if the system has the potential to replace the standard in-person evaluation, the response was slightly more positive at 4.5 of 7 (SD=2.56, Range=1–7) or 60% agreement. When asked if it is as easy to interact and be examined remotely, 68% of users agreed (Avg=5.63 of 7, SD=1.71, Range=1–7). This suggested that, while the technology shows promise, improvements must be made before it could replace in-person evaluations.

The results of the clinician specific portion of the evaluation revealed three findings. First, it was possible to evaluate patient’s arm strength using the system, with an average 6 of 7 on the Likert scale (SD=0, Range=6–6, 100% agreement). Second, patient’s limb movement was visible through the augmented reality visual system, with an average of 5.87 of 7 on the Likert scale (SD=0.35, Range=5–6, 100% agreement). Finally, the remote clinician neither agreed nor disagreed when asked if in-person and remote evaluation would provide the same results, with an average of 4 of 7 on the Likert scale (SD=0, Range=4–4, 100% neutral).

Written comments centered around three main ideas/themes (1) haptic feedback limitations, (2) visual fidelity issues, and (3) general praise for the system’s potential (Table 1).

## DISCUSSION

The current standard of care for synchronous telerehabilitation uses video conferencing software to facilitate communication between patient and provider. Telerehabilitation conference rooms set up in local clinics proximal to patients’ counties of residence provide the necessary real-time audio-video recording and playback equipment to allow confidential remote physician-patient interactions. For example, we utilize video conferencing to engage in follow up appointments with patients who live considerable distances away from specialty services available at our medical center. The conferencing system is invaluable for providing quick checkups after lengthy inpatient hospital stays, or for routine follow up weeks to months after in-person fitting and training for a new prosthetic limb. The current system is also beneficial for providing patients access to specialty services usually only available in large, densely populated cities or large medical centers. This is essential for persons with disabilities, or with limited mobility or with limited access to appropriate care.

### SUGGESTED IMPROVEMENTS

The ARTESH system attempts to take all the benefits of the current synchronous telerehabilitation system and enhances patient-provider interaction through the addition of the sense of touch and immersive 3D-video-augmented reality. Results from the evaluation of this augmented reality-based telerehabilitation system were largely positive; however, the system could be further improved. Suggested improvements fall into three main categories:

1. **Increase the haptic device’s range of motion (ROM) to allow for the full examination of all movements capable of a human shoulder**. The ideal haptic device would allow expression of the full range of motion around all joints in the upper extremity. Evaluation of movement about proximal joints would not be dependent on transmission of forces through distal structures (e.g., use of hand grip to evaluate movements of the shoulder).
2. **Find a way to evaluate patients who have issues gripping the haptic device**. This would require a haptic device with independent planes of motion about the shoulder, elbow, and wrist (with full ROM), as well as a method of securing the haptic device to the user. This problem likely requires a novel engineering solution as no currently available haptic device fully solves this issue.
3. **Improve the visual quality of tele-immersion**. Minimize stitching artifacts by use of additional 3D-cameras, or through increased post-processing of camera data. However, the synchronous nature of this live interaction would limit the amount of time for post-processing. Future updates in processing power or connectivity speeds may correct much of this issue.

### LIMITATIONS

There are some limitations to this pilot study with a convenience sample (N= 20) conducted to inform further modification of the telerehabilitation system. Because this was a qualitative study, no statistical significance can be determined. Furthermore, the study’s findings may not be generalizable to all clinical practices and locations because it requires ultra-high-speed internet with high bandwidth and speed requirements.

### FUTURE DIRECTIONS

The ARTESH system is currently not available for clinical use; it is under research development, with many potential improvements envisioned to render it more user-friendly, affordable, and clinically useful. Further studies are needed to identify what clinical scenarios are appropriate for use of this telerehabilitation system. Suggested future areas for study include conducting cost-benefit analysis; replicating similar questionnaires with larger and more diverse set of patients; quantifying productivity, ease of use, and satisfaction; and assessing clinician adoption rate and satisfaction outside the study.

## CONCLUSIONS

This pilot study aimed to evaluate the ARTESH system, an augmented-reality based telerehabilitation system with haptic feedback. It found that the system allows patients and care-providers to interact remotely, with strong potential to provide meaningful clinical encounters through auditory, visual, and tactile interactions. Results suggest that the system is effective at conveying audio-visual and touch data in real-time across 20.3 miles, and warrants further development.

## Figures and Tables

**Figure 3:**
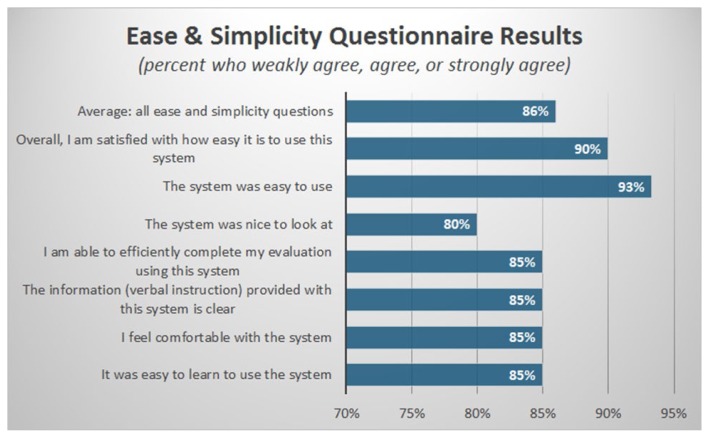
Results of “Ease and Simplicity” section of telehealth usability questionnaire reported as percent of respondents who rated > 4 on a 7-point Likert scale (corresponding to “weakly agree”, “agree”, or “strongly agree”).

**Table 1:** Written Comments

Written Comments	
Haptic Feedback Limitations	From physician volunteers: *“I can easily overpower the machine on 5/5 strength testing…It would be beneficial to have an indicator that tells me when I am overpowering the machine.” “There is not a wide difference in resistance between passive range of motion and max resistance.”* “*The range of motion allowed by the haptic feedback device seemed inadequate when compared to the full range capable by a human shoulder.”* Two post-stroke patients in the study endorsed difficulty when needing to grip the ball to maneuver the machine, noting that their weakened grip strength made operating the machine more difficult. One patient suggested a *“bigger ball for grip and a glove-like apparatus to secure the hand.”* *“Ergonomics of device knob could be more neutral for left/right setup.”* The haptic device located at patient’s site required the palm facing out for right arm evaluation, and the palm facing in for left arm evaluation. One potential solution would be to attach the haptic device to a rotating table that could accommodate both arms equally, such as the haptic device at the doctor’s site.
Visual Fidelity	*“Fuzzy video.”* “*Video quality makes it difficult to see how my patient is using the device.”* The system was later modified to allow the clinician to change at will to a hand level camera view anterior to the patient via the use of a button built into the haptic device. This helped to alleviate some of the grip ambiguity issues. However, video quality could be improved.
General Praise	From a patient: *“I think the system could be extremely useful.”* This written comment was similar to the questionnaire item discussed previously where there was 60% user agreement that “the system has the potential to replace the standard in-person evaluation.”

## Footnotes

1 Feedback loops might arise due to network delays between sites coupled with the fact that no dominant-subservient relationship existed between the haptic feedback devices (i.e., both devices had to be able to influence each other without preference).
